# On the Security of a Latin-Bit Cube-Based Image Chaotic Encryption Algorithm

**DOI:** 10.3390/e21090888

**Published:** 2019-09-12

**Authors:** Zeqing Zhang, Simin Yu

**Affiliations:** School of Automation, Guangdong University of Technology, Guangzhou 510006, China; siminyu@163.com

**Keywords:** image chaotic encryption, cryptography, Latin cube, bit cube, chosen plaintext attack

## Abstract

In this paper, the security analysis of an image chaotic encryption algorithm based on Latin cubes and bit cubes is given. The proposed algorithm adopts a first-scrambling-diffusion- second-scrambling three-stage encryption scheme. First, a finite field is constructed using chaotic sequences. Then, the Latin cubes are generated from finite field operation and used for image chaotic encryption. In addition, according to the statistical characteristics of the diffusion image in the diffusion stage, the algorithm also uses different Latin cube combinations to scramble the diffusion image for the second time. However, the generation of Latin cubes in this algorithm is independent of plain image, while, in the diffusion stage, when any one bit in the plain image changes, the corresponding number of bits in the cipher image follows the change with obvious regularity. Thus, the equivalent secret keys can be obtained by chosen plaintext attack. Theoretical analysis and experimental results indicate that only a maximum of $2.5\times \sqrt[{3}]{w\times h}+6$ plain images are needed to crack the cipher image with w×h resolution. The size of equivalent keys deciphered by the method proposed in this paper are much smaller than other general methods of cryptanalysis for similar encryption schemes.

## 1. Introduction

Image chaotic encryption algorithms have attracted some special attention in the field of information security [1,2,3,4,5,6,7]. In recent years, many image chaotic encryption schemes combined chaos theories with other technologies, such as one-time keys [8], bit-level permutation [9], DNA operations [10,11,12,13], parallel computing system [14], matrix semi-tensor product theory [15], cellular automata [16,17], neural network [18,19], Latin square or Latin cube [20,21,22], and so on, have been proposed. However, the security issues of image chaotic encryption algorithms have also attracted much attention. As a basic requirement of security, the ciphertext image of the image chaotic encryption algorithm must have good uniformity. In addition, the algorithm must have a large enough key space to resist brute force attacks. For instance, in order to show the security of the image chaotic encryption algorithm in the statistical sense, the key space analysis, statistical analysis, and differential analysis of the chaos encryption algorithm proposed in [23] and its corresponding extended algorithm are given in Sections 4 and 5 of [23], respectively. However, the high uniformity of ciphertext does not mean that the encryption algorithm has high security performance. For example, in [24], the security analysis of an image chaotic encryption algorithm proposed in [16] is given, and it is found that the generation of key stream is related to the sum of pixel values of plain images. Under the premise of satisfying the sum of pixel values of a plain image unchanged, only two pixel values of cipher image are changed corresponding to the variation of two pixel values of a plain image, which is vulnerable to differential attack. Therefore, the equivalent secret keys can be obtained by selecting 512 plain images. In [25], the cryptanalysis of a DNA encoding-based image scrambling and diffusion encryption algorithm proposed in [10] is reported to find that the scrambling algorithm is also independent of plain image, so that it can be deciphered by chosen plaintext attack. In addition, by choosing some specific plain images, the original image chaotic encryption algorithm can be simplified into scrambling-only encryption algorithm, which has been proven to be insecure [26,27]. In [28], the security analysis of an image encryption algorithm based on a compound chaotic system proposed in [29] is given, and it is pointed out that there are a large number of equivalent secret keys in the image chaotic encryption algorithm. In [30], an 8D self-synchronous and feedback-based chaotic stream cipher using the lower 8 bits of one state variable for encryption is proposed. However, in [31], most of the secret keys are successfully acquired by means of a divide and conquer attack, known plaintext attack, and a chosen ciphertext attack, respectively. In [32], the security analysis of a Latin square based image chaotic encryption algorithm proposed in [22] is given to find the security vulnerabilities both in the diffusion stage and in the scrambling stage through chosen text attack. In [33], the chosen plaintext attack is adopted for the safety performance assessment of a 1D combinatorial chaotic encryption algorithm proposed in [34]. In addition, in [35], the chosen plaintext attack is also utilized for analyzing the security of a bit cube-based image chaotic encryption algorithm proposed in [36]. In addition, some chaotic cipher designers have also discovered the importance of cryptanalysis. For example, in Section 3 of [37], the resistance to the four classic attack methods is analyzed in detail. The analysis shows that the proposed encryption algorithm has resistance to the chosen plaintext attack because it is sensitive to the initial parameters.

In 2019, an image chaotic encryption algorithm based on orthogonal Latin cubes and bit cubes is given in [20]. First, a chaotic sequence is generated by logistic mapping, and it is further arranged in ascending order to obtain its corresponding chaotic index sequence. Next, a finite field is constructed by the chaotic index sequence, and three orthogonal Latin cubes are also generated. Then, the generated three orthogonal Latin cubes are used for the first-scrambling-diffusion- second-scrambling three-stage encryption. Although the designer claims that the algorithm has passed various statistical tests, the analysis results in this paper demonstrate that the algorithm has at least two security vulnerabilities as follows:

(1) The generation of Latin cubes in this algorithm is independent of plain image.
(2) When any one bit in the plain image changes, the corresponding number of bits in the cipher image follows the change with obvious regularity.

Based on the above-mentioned security vulnerabilities, this paper adopts both chosen plaintext attack and differential attack for analyzing the safety performance for the image chaotic encryption algorithm proposed in [20]. First, a full zero plain image and multiple non-full zero plain images are selected, and the differential operation is performed between the cipher image corresponding to this full zero plain image and the cipher image corresponding to those non-full zero plain images. On the premise that the sum of bit 1 in each differential operation is even, the chaotic index sequence lx can be deciphered. Next, based on the obtained lx, and on the condition that there exists an intersection in the solutions of unary quadratic equation on finite field GF(q), the secret keys α, β, γ can be further deciphered.

The rest of the paper is organized as follows: Section 2 briefly introduces the image chaotic encryption algorithm. Section 3 presents the security analysis. Section 4 gives the steps for deciphering image chaotic encryption algorithm. Section 5 demonstrates the numerical simulation experiments. Section 6 gives some improvement suggestions for the image chaotic encryption algorithm. Finally, Section 7 concludes the paper.

## 2. Description of an Image Chaotic Encryption Algorithm

### 2.1. A Brief View of an Image Chaotic Encryption Algorithm

**Figure 1:**
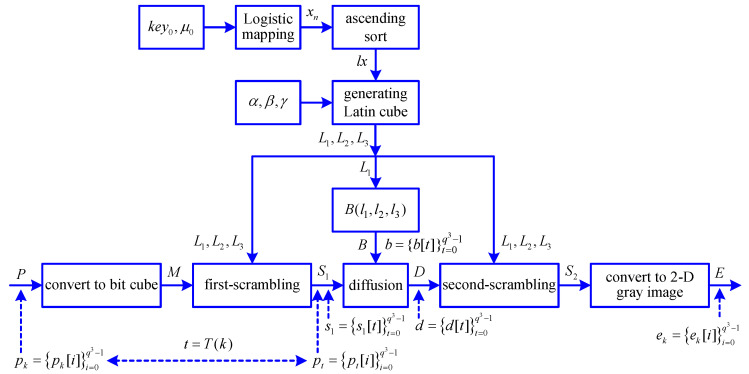
Block diagram of an image chaotic encryption algorithm.

In [20], the image chaotic encryption algorithm consists of secret keys selection, Latin cube generation, scrambling encryption, and diffusion encryption, as shown in Figure 1, where ${key}_{0}$, $\mu_{0}$, α, β, γ are the secret keys, $x_{n}$(n=0,1,2,⋯) is a chaotic sequence generated by Logistic mapping, lx is a chaotic index sequence, $L_{1}$, $L_{2}$, $L_{3}$ are three Latin cubes, *P* is a 2D plain gray image, *M* is a bit cube representation of *P*, $S_{1}$ is a first-scrambling image of *M*, *D* is a diffusion image of $S_{1}$, $S_{2}$ is a second-scrambling image of *D*, *E* is a 2D cipher gray image of $S_{2}$, and *B* is generated by $L_{1}$. When the size of the image is w×h, the length of $x_{n}$ and lx is $q=\sqrt[{3}]{8\times w\times h}$, the side length of Latin cubes and bit cubes is $q=\sqrt[{3}]{8\times w\times h}$, and the secret keys α,β,γ∈{0,1,2,⋯,q−1}. Note that an appropriate image size w×h should be selected to ensure that $q=\sqrt[{3}]{8\times w\times h}=2\times \sqrt[{3}]{w\times h}$ is an even number. In Figure 1, $L_{1},\;L_{2},\;L_{3}\in \left\{0,1,2,\cdots ,q-1\right\}$ are Latin cubes, $M,\;S_{1},\;D,\;S_{2},\;B\in \left\{0,1\right\}$ are bit cubes, *P* is a 2D plain gray image, *E* is a 2D cipher gray image, $p_{k},\;p_{t},\;s_{1},\;b,\;d,\;e_{k}\in \left\{0,1\right\}$ are 1D bit sequences corresponding to $P,\;S_{1},\;B,\;D,\;E$, and t=T(k) is a position scrambling rule corresponding to the first-scrambling stage.

### 2.2. Logistic Map

According to Figure 1, the chaotic sequence is generated through logistic mapping, given by

(1) $$x_{n+1}=\mu x_{n}\left(1-x_{n}\right),$$

where n=0,1,2,⋯, $x_{n}\in \left(0,1\right)$, 0≤μ≤4. When μ>3.573815, Equation (1) is chaotic.

### 2.3. Generation of Latin Cubes

**Figure 2:**
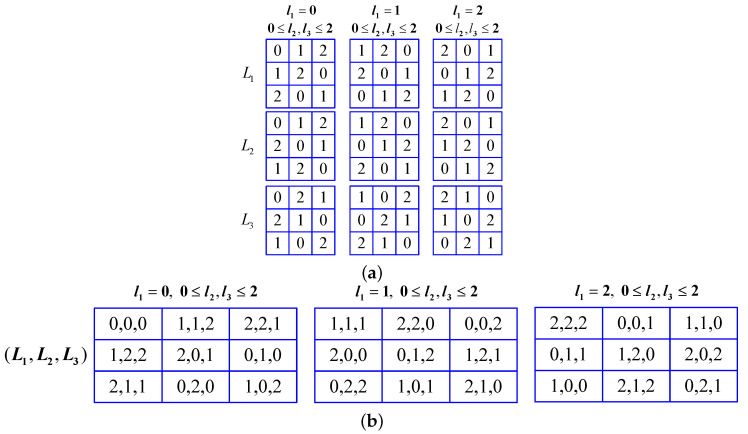
Three orthogonal Latin cubes and the corresponding triple tuple when q=3. (**a**) three orthogonal Latin cubes; (**b**) the corresponding triple tuple.

Let the side length of $L_{1}$, $L_{2}$, $L_{3}$ be $q=\sqrt[{3}]{8\times w\times h}$, where *q* is an even number. For a given $\left(l_{1},l_{2},l_{3}\right)$, one gets $L_{1}\left(l_{1},l_{2},l_{3}\right)=\psi_{1}$, $L_{2}\left(l_{1},l_{2},l_{3}\right)=\psi_{2}$, $L_{3}\left(l_{1},l_{2},l_{3}\right)=\psi_{3}$, $0\leq \psi_{1},\psi_{2},\psi_{3}\leq q-1$. If $\left(l_{1},l_{2},l_{3}\right)\neq \left(l_{1}^{\prime },l_{2}^{\prime },l_{3}^{\prime }\right)$, $\left(\psi_{1},\psi_{2},\psi_{3}\right)\neq \left(\psi_{1}^{\prime },\psi_{2}^{\prime },\psi_{3}^{\prime }\right)$, then $L_{1},L_{2},L_{3}$ are orthogonal to each other [38]. When q=3, one gets three orthogonal Latin cubes, as shown in Figure 2a, and the corresponding triple tuple is shown in Figure 2b, respectively.

The algorithm for generating Latin cubes proposed in [20] is implemented by replacing the ordered set {0,1,2,…,q} in the generation method proposed in [38] with the chaotic index sequence lx. The detailed steps for generating three orthogonal Latin cubes by means of a finite field are in Algorithm 1.

**Algorithm 1** Steps for Generation of Latin Cubes.**Input:**    Secret keys ${key}_{0},\;\mu_{0},\;\alpha ,\;\beta ,\;\gamma$; Side length $q=\sqrt[{3}]{8\times w\times h}$; **Output:**    Three orthogonal Latin cubes $L_{1}$, $L_{2}$ and $L_{3}$;
1:Generate the chaotic sequence $x=\{x_{0},x_{1},\ldots ,x_{q-1}\}$ by using Logistic mapping.2:Obtain the corresponding chaotic index sequence $lx=\{c_{0},c_{1},\cdots ,c_{i},\cdots ,c_{q-1}\}$ by arranging $x=\{x_{0},x_{1},\ldots ,x_{q-1}\}$ in ascending order, where $0\leq c_{i},\;i\leq q-1$, satisfying $lx\left[i\right]=c_{i}$. Note that the chaotic index sequence lx can only be determined after the sequence value $c_{i}$ and the sequence number *i* are simultaneously obtained. When the sequence value $c_{i}$ is obtained, but the sequence number *i* is uncertain, the general form of the chaotic index sequence lx is in the form of
(2)$$lx=\{c_{i_{0}},c_{i_{1}},\cdots ,c_{i_{k}},\cdots ,c_{i_{q-1}}\},$$
where $0\leq c_{i_{k}}\;i_{k}\leq q-1$, $i_{0}\neq i_{1}\neq \cdots \neq i_{k}\neq \cdots \neq i_{q-1}$, $lx\left[i_{k}\right]=c_{i_{k}}$. In the following, ξ or $\xi^{\prime }$ denotes the sequence value and $i_{\xi }$ or $i_{\xi^{\prime }}^{\prime }$ denotes the sequence number in Equation (2), respectively.3:Construct a finite field by using chaotic index sequence lx, and then one gets the orthogonal Latin cubes on the finite field, given by
(3)$$\left\{\begin{array}{c} L_{1}\left(l_{1},l_{2},l_{3}\right)=\alpha^{2}\times c_{l_{1}}+\alpha \times c_{l_{2}}+c_{l_{3}},\\ L_{2}\left(l_{1},l_{2},l_{3}\right)=\beta^{2}\times c_{l_{1}}+\beta \times c_{l_{2}}+c_{l_{3}},\\ L_{3}\left(l_{1},l_{2},l_{3}\right)=\gamma^{2}\times c_{l_{1}}+\gamma \times c_{l_{2}}+c_{l_{3}}, \end{array}\right.$$
where “+” denotes addition operation on the finite field, “×” denotes multiplication operation on the finite field, α,β,γ∈lx, $c_{l_{1}}$, $c_{l_{2}}$, $c_{l_{3}}$ are sequence values of lx.4:**return**$L_{1}$, $L_{2}$, $L_{3}$.


### 2.4. Steps for Image Chaotic Encryption

According to Figure 1, and taking a plain gray image with 512×512 resolution as an example, one has $q=\sqrt[{3}]{512\times 512\times 8}=128$. The steps for image chaotic encryption are in Algorithm 2.

**Algorithm 2** Steps for Image Chaotic Encryption.**Input:**   Secret keys ${key}_{0},\;\mu_{0},\;\alpha ,\;\beta ,\;\gamma$; Plaintext image *P*; **Output:**   Ciphertxet image *E*;
1:Convert the 2D plain gray image *P* into the bit cube *M*;2:Obtain three orthogonal Latin cubes $L_{1}$, $L_{2}$, $L_{3}$ by Algorithm 1;3:Scramble bit cube *M* by using three orthogonal Latin cubes $L_{1},L_{2},L_{3}$, and get the corresponding first-scrambling image $S_{1}$ in the form of bit cube, such that
(4)$$S_{1}\left(l_{1},l_{2},l_{3}\right)=M\left(L_{1}\left(l_{1},l_{2},l_{3}\right),L_{2}\left(l_{1},l_{2},l_{3}\right),L_{3}\left(l_{1},l_{2},l_{3}\right)\right).$$4:Obtain the diffusion bit cube $B\left(l_{1},l_{2},l_{3}\right)$ by using Latin cube $L_{1}$, given by
(5)$$B\left(l_{1},l_{2},l_{3}\right)=\left\{\begin{array}{ll} 0, & \mathrm{if}\;L_{1}\left(l_{1},l_{2},l_{3}\right)\geq 64,\\ 1, & \mathrm{if}\;L_{1}\left(l_{1},l_{2},l_{3}\right)<64. \end{array}\right.$$Then, get the diffusion 1D bit sequence b[t] corresponding to diffusion bit cube $B(l_{1},l_{2},l_{3})$ as
(6)$$b\left[t\right]=B\left(\left\lfloor t/128^{2}\right\rfloor ,\;\left\lfloor t/128\right\rfloor \%128,\;t\%128\right),$$
where $t\in \{0,1,2,\cdots ,q^{3}-1\}$, $\left\lfloor \cdot \right\rfloor$ is a round down operation, and “%” is a modulo operation.5:Convert $S_{1}\left(l_{1},l_{2},l_{3}\right)$ into the 1D bit sequence $s_{1}\left[t\right]$ as
(7)$$s_{1}\left[t\right]=S_{1}\left(\left\lfloor t/128^{2}\right\rfloor ,\;\left\lfloor t/128\right\rfloor \%128,\;t\%128\right).$$Then, get the 1D bit sequence d[t] by using $s_{1}\left[t\right]$ and b[t] as
(8)$$d\left[t\right]=s_{1}\left[t\right]⊕d\left[t-1\right]⊕b\left[t\right],$$
where $0\leq t\leq 128^{3}-1$, d[−1]=0, “⊕” denotes bitwise exclusive or operation.6:Calculate $G\left(d\right)=\left(\sum_{i=0}^{q^{3}-1}d[i]\right)$, and convert the 1D bit sequence d[t] into the bit cube $D(l_{1},l_{2},l_{3})$. Then, get the bit cube $S_{2}\left(l_{1},\;l_{2},\;l_{3}\right)$ by utilizing $D(l_{1},\;l_{2},\;l_{3})$, such that
(9)$$S_{2}\left(l_{1},\;l_{2},\;l_{3}\right)=\left\{\begin{array}{ll} D\left(L_{2}\left(l_{1},l_{2},l_{3}\right),L_{3}\left(l_{1},l_{2},l_{3}\right),L_{1}\left(l_{1},l_{2},l_{3}\right)\right), & \left(G\left(d\right)\%2=0\right),\\ D\left(L_{3}\left(l_{1},l_{2},l_{3}\right),L_{1}\left(l_{1},l_{2},l_{3}\right),L_{2}\left(l_{1},l_{2},l_{3}\right)\right), & (G(d)\%2=1), \end{array}\right.$$
where G(d)%2∈{0,1} denotes the modular 2 operation on G(d).7:Convert the bit cube $S_{2}\left(l_{1},\;l_{2},\;l_{3}\right)$ into the 2D cipher gray image *E* with 512×512 resolution.8:**return***E*.


**Figure 3:**
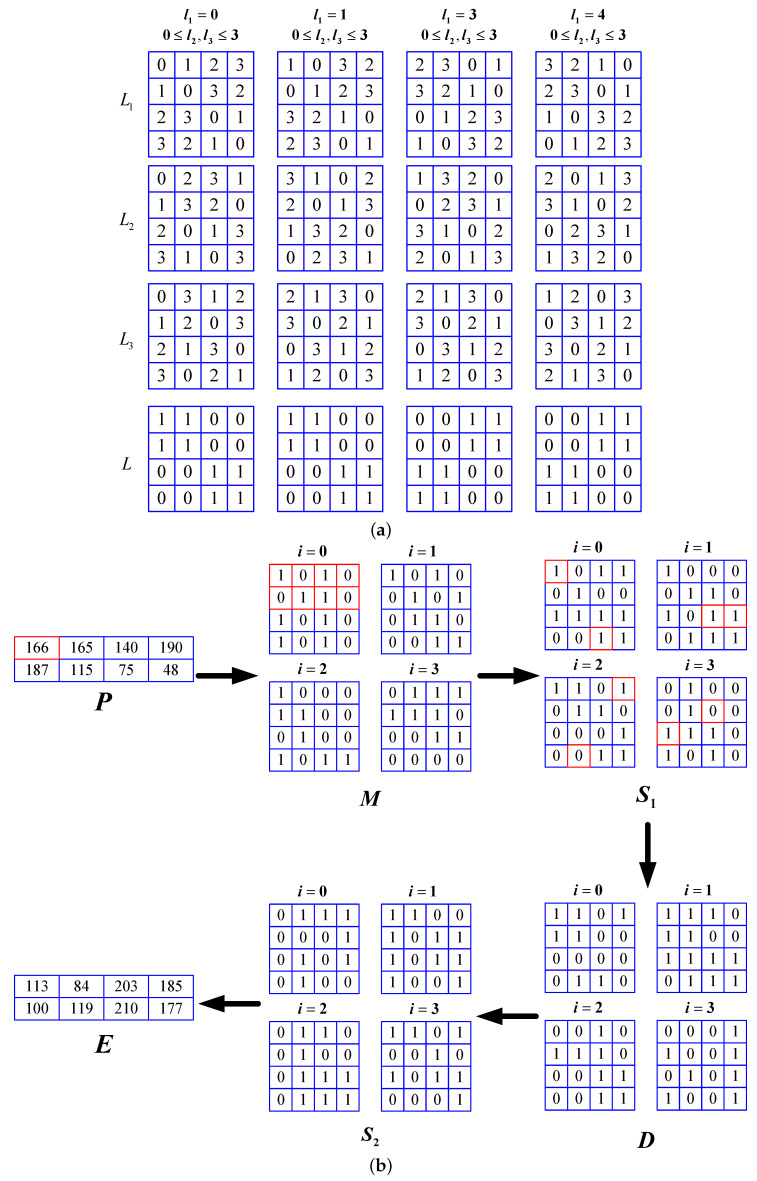
An example of encrypting a gray image with 2×4 resolution. (**a**) three orthogonal Latin cubes and the corresponding bit cubes *L* used for encryption; (**b**) the encryption process.

An example of encrypting a gray image with 2×4 resolution using the original encryption algorithm is shown in Figure 3. Figure 3a shows the three Latin cubes and the corresponding bit cubes *L* used for encryption. Figure 3b shows the encryption process. The numbers in the cells of *P* and *E* represent pixel values, and the bit values are represented in the cells of $S_{1}$, *B*, and $S_{2}$. The red cells in *M* indicate that they are bit representations of the red cell corresponding to *P*, i.e., the binary representation of 166 is ${\left(10100110\right)}_{2}$.

## 3. Security Analysis

According to Figure 1, it is found that the generation of three orthogonal Latin cubes $L_{1},\;L_{2},\;L_{3}$ is not related to the plain image. When the secret keys are given, the three orthogonal Latin cubes $L_{1},\;L_{2},\;L_{3}$ remain unchanged for different input plain images, which are provided a prerequisite for chosen plaintext attack. Therefore, one can decipher the equivalent secret keys lx,α,β,γ corresponding to the original secret keys ${key}_{0},\;\mu_{0},\;\alpha ,\;\beta ,\;\gamma$.

### 3.1. Analysis of Chaotic Index Sequence lx

#### 3.1.1. Relation between the First-Scrambling Image $S_{1}$ and the Plain Image *M*

**Proposition** **1.** *Suppose that M is the bit cube representation of P; $S_{1}$ is the first-scrambling image of M. The relationship between M and $S_{1}$ satisfies $S_{1}\left(i_{0},i_{0},i_{\xi }\right)=M\left(\xi ,\xi ,\xi \right)$, where $lx[i_{0}]=0$, $lx[i_{\xi }]=\xi$, $i_{0},\;\xi \in \left\{0,\;1,\;2,\;\cdots ,\;q-1\right\}$, $i_{0}$ denotes the sequence number corresponding to the sequence value 0, and $i_{\xi }$ denotes the sequence number corresponding to the sequence value ξ.*

**Proof.**  Let $l_{1}=l_{2}=i_{0},\;l_{3}=i_{\xi }$, and substitute them into Equation (4), then, one gets

(10) $$S_{1}\left(i_{0},i_{0},i_{\xi }\right)=M\left(L_{1}\left(i_{0},i_{0},i_{\xi }\right),L_{2}\left(i_{0},i_{0},i_{\xi }\right),L_{3}\left(i_{0},i_{0},i_{\xi }\right)\right).$$

In addition, let $l_{1}=l_{2}=i_{0},\;l_{3}=i_{\xi }$, and substitute them into Equation (3), then, one gets

(11) $$\left\{\begin{array}{c} L_{1}\left(i_{0},i_{0},i_{\xi }\right)=\alpha^{2}\times c_{i_{0}}+\alpha \times c_{i_{0}}+c_{i_{\xi }},\\ L_{2}\left(i_{0},i_{0},i_{\xi }\right)=\beta^{2}\times c_{i_{0}}+\beta \times c_{i_{0}}+c_{i_{\xi }},\\ L_{3}\left(i_{0},i_{0},i_{\xi }\right)=\gamma^{2}\times c_{i_{0}}+\gamma \times c_{i_{0}}+c_{i_{\xi }}. \end{array}\right.$$

Since $lx[i_{0}]=0$, $lx[i_{\xi }]=\xi$, one has $lx\left[i_{0}\right]=c_{i_{0}}=0$, $lx\left[i_{\xi }\right]=c_{i_{\xi }}=\xi$. In addition, substituting $c_{i_{0}}=0$ and $c_{i_{\xi }}=\xi$ into Equation (11), one gets

(12) $$L_{1}\left(i_{0},i_{0},i_{\xi }\right)=L_{2}\left(i_{0},i_{0},i_{\xi }\right)=L_{3}\left(i_{0},i_{0},i_{\xi }\right)=\xi .$$

In addition, substituting Equation (12) into Equation (10), it follows that $S_{1}\left(i_{0},i_{0},i_{\xi }\right)=M\left(\xi ,\xi ,\xi \right)$ holds. The proof is finished. □

#### 3.1.2. The First Case for Analysis of Chaotic Index Sequence lx

Suppose that the 1D bit sequence corresponding to plain image $P_{0}$ is ${\left\{p_{0}\left[i\right]\right\}}_{i=0}^{q^{3}-1}={\left\{0\right\}}_{i=0}^{q^{3}-1}$, the cipher image corresponding to plain image $P_{0}$ is $E_{0}$, the 1D bit sequence corresponding to cipher image $E_{0}$ is ${\left\{e_{0}\left[i\right]\right\}}_{i=0}^{q^{3}-1}$, and the 1D bit sequence corresponding to plain image $P_{k}$ is ${\left\{p_{k}\left[i\right]\right\}}_{i=0}^{q^{3}-1}$, where $p_{k}\left[i\right]$ is given by

(13) $$p_{k}\left[i\right]=\left\{\begin{array}{cc} 1, & \mathrm{if}\;i=k,\\ 0, & \mathrm{if}\;i\neq k. \end{array}\right.$$

In addition, suppose that the cipher image corresponding to plain image $P_{k}$ is $E_{k}$, the 1D bit sequence corresponding to cipher image $E_{k}$ is ${\left\{e_{k}\left[i\right]\right\}}_{i=0}^{q^{3}-1}$, the 1D bit sequence corresponding to plain image $P_{k_{1}k_{2}}=P_{k_{1}}⊕P_{k_{2}}$ is ${\left\{p_{k_{1}k_{2}}\left[i\right]\right\}}_{i=0}^{q^{3}-1}={\left\{p_{k_{1}}\left[i\right]⊕p_{k_{2}}\left[i\right]\right\}}_{i=0}^{q^{3}-1}$, the cipher image corresponding to plain image $P_{k_{1}k_{2}}$ is $E_{k_{1}k_{2}}$, the 1D bit sequence corresponding to cipher image $E_{k_{1}k_{2}}$ is ${\left\{e_{k_{1}k_{2}}\left[i\right]\right\}}_{i=0}^{q^{3}-1}$, the 1D bit sequence corresponding to plain image $P_{k_{1}k_{2}k_{3}}=P_{k_{1}}⊕P_{k_{2}}⊕P_{k_{3}}$ is ${\left\{p_{k_{1}k_{2}k_{3}}\left[i\right]\right\}}_{i=0}^{q^{3}-1}={\left\{p_{k_{1}}\left[i\right]⊕p_{k_{2}}\left[i\right]⊕p_{k_{3}}\left[i\right]\right\}}_{i=0}^{q^{3}-1}$, the cipher image corresponding to $P_{k_{1}k_{2}k_{3}}$ is $E_{k_{1}k_{2}k_{3}}$, and the 1D bit sequence corresponding to $E_{k_{1}k_{2}k_{3}}$ is ${\left\{e_{k_{1}k_{2}k_{3}}\left[i\right]\right\}}_{i=0}^{q^{3}-1}$.

**Proposition** **2.** *Suppose that the cipher image corresponding to plain image $P_{k}$ is $E_{k}$, the 1D bit sequence corresponding to cipher image $E_{k}$ is ${\left\{e_{k}\left[i\right]\right\}}_{i=0}^{q^{3}-1}$, the cipher image corresponding to plain image $P_{0}$ is $E_{0}$, and the 1D bit sequence corresponding to cipher image $E_{0}$ is ${\left\{e_{0}\left[i\right]\right\}}_{i=0}^{q^{3}-1}$. A differential operation is performed in the form of $\sum_{i=0}^{q^{3}-1}\left(e_{0}\left[i\right]⊕e_{k}\left[i\right]\right)=q^{3}-m_{k,0}$, in which $e_{k}\left[i_{l}\right]=e_{0}\left[i_{l}\right]\;(l=1,2,\cdots ,m_{k,0};\;i_{l}\in \left\{0,1,2,\cdots ,q^{3}-1\right\})$, $q^{3}$ is an even number. If $\left(q^{3}-m_{k,0}\right)\%2=q^{3}\%2-m_{k,0}\%2=m_{k,0}\%2=0$, then $T\left(k\right)=m_{k,0}$ holds, where T(k) denotes the position scrambling rule in the first-scrambling stage, k denotes the position of the k-th bit before the first-scrambling of plain image, and T(k) denotes the position of k-th bit after the first-scrambling of plain image.*

**Proof.**  According to Equation (6), the relationship between the coordinates $\left(l_{1},l_{2},l_{3}\right)$ of bit cube $B(l_{1},l_{2},l_{3})$ and the position *t* of 1D bit sequence b[t] corresponding to $B(l_{1},l_{2},l_{3})$ is given by

(14) $$\left\{\begin{array}{c} l_{1}=\left\lfloor t/q^{2}\right\rfloor =\left\lfloor t/128^{2}\right\rfloor ,\\ l_{2}=\left\lfloor t/q\right\rfloor \%q=\left\lfloor t/128\right\rfloor \%128,\\ l_{3}=t\%q=t\%128. \end{array}\right.$$

On the other hand, the relationship between the coordinates (ξ,ξ,ξ) of bit cube M(ξ,ξ,ξ) and the position *k* of 1D bit sequence $p_{k}\left[i\right]$ in Equation (13) is given by

(15) $$k=\xi (q^{2}+q+1).$$

Thus, the relationship between the position of t-th bit after the first-scrambling of plain image and the position of k-th bit before the first-scrambling of plain image is given by

(16) $$t=T\left(k\right)=T(\xi \left(q^{2}+q+1\right)).$$

(1) Consider the first-scrambling stage. In the first-scrambling stage, only change the bit position, but the bit value should remain unchanged. Suppose that the input 1D bit sequence corresponding to plain image $P_{k}$ is $p_{k}$, after the first-scrambling of plain image, the corresponding output 1D bit sequence is $p_{t}$. According to Equation (16), the relationship between position *t* and *k* satisfies t=T(k). In particular, if the input 1D bit sequence corresponding to plain image $P_{0}$ is $p_{0}={\left\{p_{0}\left[i\right]\right\}}_{i=0}^{q^{3}-1}={\left\{0\right\}}_{i=0}^{q^{3}-1}$, after the first-scrambling of plain image, the corresponding output 1D bit sequence is $p_{t}={\left\{p_{t}\left[i\right]\right\}}_{i=0}^{q^{3}-1}$, then one has $p_{t}=p_{0}={\left\{0\right\}}_{i=0}^{q^{3}-1}$. (2) Consider the diffusion stage. Take the output 1D bit encryption sequence ${\left\{p_{o}\left[i\right]\right\}}_{i=0}^{q^{3}-1}$ in the first-scrambling stage as the input 1D bit sequence in the diffusion stage. According to Equation (8), diffuse ${\left\{p_{o}\left[i\right]\right\}}_{i=0}^{q^{3}-1}$ by using the diffusion 1D bit sequence ${\left\{b\left[i\right]\right\}}_{i=0}^{q^{3}-1}$, obtain the corresponding output ${\left\{d_{o}\left[i\right]\right\}}_{i=0}^{128^{3}-1}$ in the diffusion stage. By substituting $s_{1}\left[i\right]=p_{o}\left[i\right]=0$ into Equation (8), one has

(17) $$\left\{\begin{array}{cc} d_{o}\left[0\right] & =p_{o}\left[0\right]⊕d_{o}\left[-1\right]⊕b\left[0\right]=0⊕0⊕b\left[0\right]=b\left[0\right],\\ d_{o}\left[1\right] & =p_{o}\left[1\right]⊕d_{o}\left[0\right]⊕b\left[1\right]=0⊕d_{o}\left[0\right]⊕b\left[1\right]=b\left[0\right]⊕b\left[1\right],\\ d_{o}\left[2\right] & =p_{o}\left[2\right]⊕d_{o}\left[1\right]⊕b\left[2\right]=0⊕d_{o}\left[1\right]⊕b\left[2\right]=b\left[0\right]⊕b\left[1\right]⊕b\left[2\right],\\ \cdots & \\ d_{o}\left[i\right] & =b\left[0\right]⊕b\left[1\right]⊕b\left[2\right]\cdots ⊕b\left[i\right], \end{array}\right.$$

where $i=0,1,2,\cdots ,q^{3}-1$, $d_{0}\left[-1\right]=0$. Similarly, take the output 1D bit encryption sequence ${\left\{p_{t}\left[i\right]\right\}}_{i=0}^{q^{3}-1}$ in the first-scrambling stage as the input 1D bit sequence in the diffusion stage. According to Equation (8), diffuse ${\left\{p_{t}\left[i\right]\right\}}_{i=0}^{q^{3}-1}$ by using the diffusion 1D bit sequence ${\left\{b\left[i\right]\right\}}_{i=0}^{q^{3}-1}$ and obtain the corresponding output ${\left\{d_{t}\left[i\right]\right\}}_{i=0}^{128^{3}-1}$ in the diffusion stage. By substituting $s_{1}\left[i\right]=p_{t}\left[i\right]$ into Equation (8), and also by utilizing Equation (17), one has

(18) $$\left\{\begin{array}{c} d_{t}\left[0\right]=p_{t}\left[0\right]⊕d_{t}\left[-1\right]⊕b\left[0\right]=0⊕0⊕b\left[0\right]=d_{o}\left[0\right],\\ d_{t}\left[1\right]=p_{t}\left[1\right]⊕d_{t}\left[0\right]⊕b\left[1\right]=0⊕d_{o}\left[0\right]⊕b\left[1\right]=0⊕b\left[0\right]⊕b\left[1\right]=d_{o}\left[1\right],\\ \cdots \\ d_{t}\left[t\right]=p_{t}\left[t\right]⊕d_{t}\left[t-1\right]⊕b\left[t\right]=1⊕d_{o}\left[t-1\right]⊕b\left[t\right]=1⊕d_{o}\left[t\right]=\bar{d_{o}\left[t\right]},\\ d_{t}\left[t+1\right]=p_{t}\left[t+1\right]⊕d_{t}\left[t\right]⊕b\left[t+1\right]=0⊕d_{t}\left[t\right]⊕b\left[t+1\right]=\bar{d_{o}\left[t\right]}⊕b\left[t+1\right]=\bar{d_{o}\left[t+1\right]},\\ \cdots \\ d_{t}\left[i\right]=p_{t}\left[i\right]⊕d_{t}\left[i-1\right]⊕b\left[i\right]=1⊕d_{o}\left[i-1\right]⊕b\left[i\right]=\bar{d_{o}\left[i\right]}, \end{array}\right.$$

where $d_{t}\left[-1\right]=0$. According to Equation (18), one has

(19) $$\left\{\begin{array}{cc} d_{t}\left[i\right]=d_{o}\left[i\right] & \;(0\leq i<t),\\ d_{t}\left[i\right]=\bar{d_{o}\left[i\right]} & \;(t\leq i\leq (q^{3}-1)), \end{array}\right.$$

where $\bar{d_{o}\left[i\right]}$ denotes the bitwise NOT of $d_{o}\left[i\right]$. (3) Consider the second-scrambling stage. Take the output 1D bit encryption sequences ${\left\{d_{o}\left[i\right]\right\}}_{i=0}^{q^{3}-1}$ and ${\left\{d_{t}\left[i\right]\right\}}_{i=0}^{q^{3}-1}$ in the diffusion stage as the input 1D bit sequences in the second-scrambling stage, calculate $G\left(d_{0}\right)=\left(\sum_{i=0}^{q^{3}-1}d_{0}\left[i\right]\right)$, $G\left(d_{t}\right)=\left(\sum_{i=0}^{q^{3}-1}d_{t}\left[i\right]\right)$, respectively. If t%2=0 in Equation (19) holds, then it follows that

(20) $$G\left(d_{t}\right)\%2=G\left(d_{0}\right)\%2.$$

According to Equation (9) with Equation (20), it is noted that the same scrambling rule for ${\left\{d_{o}\left[i\right]\right\}}_{i=0}^{q^{3}-1}$ and ${\left\{d_{t}\left[i\right]\right\}}_{i=0}^{q^{3}-1}$ is used in the second-scrambling stage. By comparing the first equation $d_{t}\left[i\right]=d_{o}\left[i\right]\;(0\leq i<t)$ of Equation (19) with $e_{k}\left[i_{l}\right]=e_{0}\left[i_{l}\right]\;(l=1,2,\cdots ,m_{k,0};\;i_{l}\in \left\{0,1,2,\cdots ,q^{3}-1\right\})$, it follows that $t=m_{k,0}$. Then, according to Equation (16), $T\left(k\right)=m_{k,0}$ holds. The proof is finished. □

Based on Proposition 1, one has $S_{1}\left(i_{0},i_{0},i_{\xi }\right)=M\left(\xi ,\xi ,\xi \right)$, where ξ∈{0,1,2,⋯,q−1} is the sequence value of chaotic index sequence lx, $i_{\xi }$ is the sequence number of lx. However, even though ξ is given, since $S_{1}\left(i_{0},i_{0},i_{\xi }\right)$ is the first-scrambling result of bit cube M(ξ,ξ,ξ), but the scrambling rule T(·) is unknown beforehand, the sequence numbers $i_{0}$ and $i_{\xi }$ cannot be directly available. Thus, Proposition 2 is needed to obtain the specific numbers $i_{0}$ and $i_{\xi }$.

Based on Proposition 2, suppose that the input plain image $M(l_{1},l_{2},l_{3})$ is given by

(21) $$M\left(l_{1},l_{2},l_{3}\right)=\left\{\begin{array}{cc} 1, & \mathrm{if}\;l_{1}=l_{2}=l_{3}=\xi ,\\ 0, & \mathrm{otherwise}, \end{array}\right.$$

where ξ∈{0,1,⋯,q−1}. Based on Equation (15) with Equation (21), one has $k=\xi \cdot (q^{2}+q+1)$. Next, one obtains $m_{k,0}$ by a chosen plaintext attack. If $m_{k,0}\%2=0$ holds, then the same scrambling rule is used for $d_{0}$ and $d_{t}$ in the second-scrambling stage, such that $T\left(k\right)=m_{k,0}=t$. Finally, according to Equation (14), it follows that

(22) $$\left\{\begin{array}{cc} \; & i_{0}=\left\lfloor t/q^{2}\right\rfloor =\left\lfloor T\left(\xi \cdot \left(q^{2}+q+1\right)\right)/q^{2}\right\rfloor =\left\lfloor T\left(k\right)/q^{2}\right\rfloor =\left\lfloor m_{k,0}/q^{2}\right\rfloor ,\\ \; & i_{\xi }=t\%q=T\left(\xi \cdot \left(q^{2}+q+1\right)\right)\%q=T\left(k\right)\%q=m_{k,0}\%q. \end{array}\right.$$

**Figure 4:**
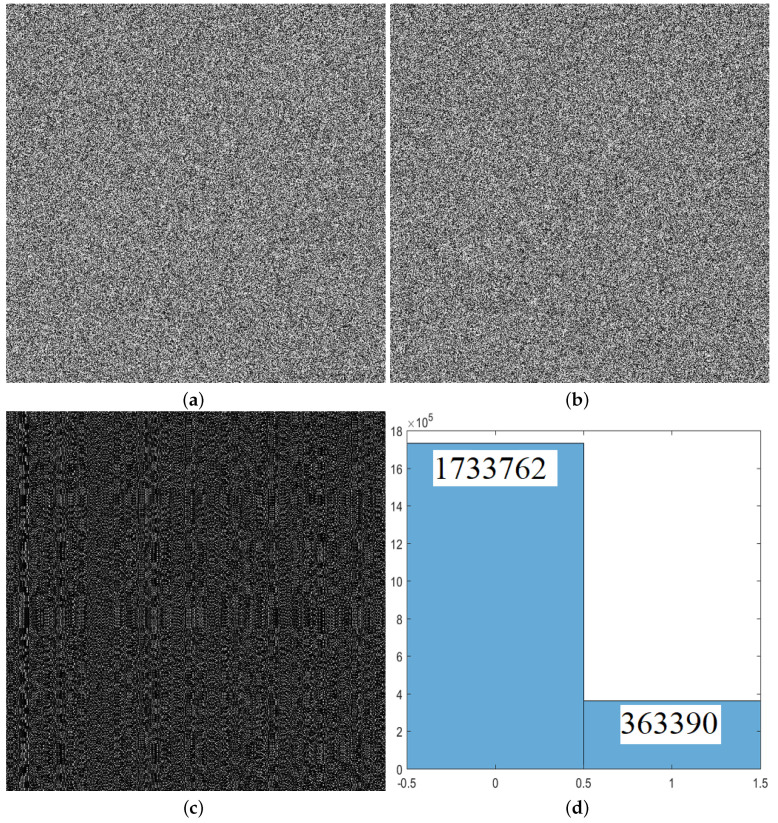
An example of Proposition 2. (**a**) the ciphertext corresponding to the grayscale image lena; (**b**) the corresponding ciphertext image after changing the bit at the bit-cube coordinates (6, 6, 6) of lena; (**c**) the bitwise exclusive or result between Figure 4a,b; (**d**) the bit statistical histogram of Figure 4c.

An example of Proposition 2 is as in Figure 4. Figure 4a shows the ciphertext corresponding to the grayscale image lena. Figure 4b shows the corresponding ciphertext image after changing the bit at the bit-cube coordinates (6, 6, 6) of lena. Figure 4c is a bitwise exclusive or result between Figure 4a,b. Figure 4d is a bit statistical histogram of Figure 4c.

The difference between the two plaintexts is only 1 bit. It can be found from Figure 4d that the number of identical bits between their corresponding ciphertexts is 1,733,762, which is an even number. Substituting $m_{k,0}=1,733,762$, ξ=6, and q=128 into Equation (22) yields $i_{0}=105$ and $i_{6}=2$.

#### 3.1.3. The Second Case for Analysis of Chaotic Index Sequence lx

If $m_{k,0}\%2\neq 0$, the above-mentioned method is no longer available, which needs to be further consideration.

**Corollary** **1.** *Supposing that the cipher image corresponding to plain image $P_{k_{1}k_{2}}=P_{k_{1}}⊕P_{k_{2}}\;\left(k_{1}\neq k_{2}\right)$ is $E_{k_{1}k_{2}}$, the 1D bit sequence corresponding to $E_{k_{1}k_{2}}$ is ${\left\{e_{k_{1}k_{2}}\left[i\right]\right\}}_{i=0}^{q^{3}-1}$, the cipher image corresponding to plain image $P_{0}$ is $E_{0}$, the 1D bit sequence corresponding to $E_{0}$ is ${\left\{e_{0}\left[i\right]\right\}}_{i=0}^{q^{3}-1}$. A differential operation is performed in the form of $\sum_{i=0}^{q^{3}-1}\left(e_{0}\left[i\right]⊕e_{k_{1}k_{2}}\left[i\right]\right)=m_{k_{1}k_{2},0}$, in which $e_{k}\left[i_{l}\right]\neq e_{0}\left[i_{l}\right]\;(l=1,2,\cdots ,m_{k_{1}k_{2},0};\;i_{l}\in \left\{0,1,2,\cdots ,q^{3}-1\right\})$. If $m_{k_{1}k_{2},0}\%2=0$, then $\left|T\left(k_{1}\right)-T\left(k_{2}\right)\right|=m_{k_{1}k_{2},0}$ holds. In addition, if $\left|T\left(k_{1}\right)-T\left(k_{2}\right)\right|\%2=0$, then $m_{k_{1}k_{2},0}=\left|T\left(k_{1}\right)-T\left(k_{2}\right)\right|$ also holds.*

**Corollary** **2.** *Suppose that the cipher image corresponding to plain image $P_{k_{1}k_{2}k_{3}}=P_{k_{1}}⊕P_{k_{2}}⊕P_{k_{3}}\;\left(k_{1}\neq k_{2}\neq k_{3}\right)$ is $E_{k_{1}k_{2}k_{3}}$, the 1D bit sequence corresponding to $E_{k_{1}k_{2}k_{3}}$ is ${\left\{e_{k_{1}k_{2}k_{3}}\left[i\right]\right\}}_{i=0}^{q^{3}-1}$, the cipher image corresponding to plain image $P_{0}$ is $E_{0}$, the 1D bit sequence corresponding to $E_{0}$ is ${\left\{e_{0}\left[i\right]\right\}}_{i=0}^{q^{3}-1}$. A differential operation is performed in the form of $\sum_{i=0}^{q^{3}-1}\left(e_{0}\left[i\right]⊕e_{k_{1}k_{2}k_{3}}\left[i\right]\right)=q^{3}-m_{k_{1}k_{2}k_{3},0}$, in which $e_{k_{1}k_{2}k_{3}}\left[i_{l}\right]=e_{0}\left[i_{l}\right]\;(l=1,2,\cdots ,m_{k_{1}k_{2}k_{3},0};\;i_{l}\in \left\{0,1,2,\cdots ,q^{3}-1\right\})$, $q^{3}$ is an even number. If $\left(q^{3}-m_{k_{1}k_{2}k_{3},0}\right)\%2=q^{3}\%2-m_{k_{1}k_{2}k_{3},0}\%2=m_{k_{1}k_{2}k_{3},0}\%2=0$, then $T\left(k_{1}\right)+T\left(k_{2}\right)-T\left(k_{3}\right)=m_{k_{1}k_{2}k_{3},0}$ holds, where $T\left(k_{1}\right)<T\left(k_{3}\right)<T\left(k_{2}\right)$ or $T\left(k_{1}\right)>T\left(k_{3}\right)>T\left(k_{2}\right)$. In addition, if $\left[T\left(k_{1}\right)+T\left(k_{2}\right)-T\left(k_{3}\right)\right]\%2=0$, then $m_{k_{1}k_{2}k_{3},0}=T\left(k_{1}\right)+T\left(k_{2}\right)-T\left(k_{3}\right)$ also holds.*

Suppose that the set of all sequence values corresponding to the chaotic index sequence lx is $\Omega =\{\xi_{i_{1}},\;\xi_{i_{2}},\;\cdots ,\;\xi_{i_{q/2}},\;\xi_{i_{1}^{\prime }}^{\prime },\;\xi_{i_{2}^{\prime }}^{\prime },\;\cdots ,\;\xi_{i_{q/2}^{\prime }}^{\prime }\}$. Let $\Psi =\{\xi_{i_{1}},\xi_{i_{2}},\cdots ,\xi_{i_{q/2}}\}$ be the set of sequence value ξ corresponding to sequence number $i_{\xi }$, where $i_{\xi }$ is obtained by using Equation (22). The relationship among ξ,k,t is $k=\xi (q^{2}+q+1)$ and $t=T\left(k\right)=T(\xi \cdot \left(q^{2}+q+1\right))$. For ∀ξ∈Ψ, $m_{k,0}\%2=0$ and $t=m_{k,0}$ hold. Similarly, let $\Psi^{\prime }=\left\{\xi_{i_{1}^{\prime }}^{\prime },\;\xi_{i_{2}^{\prime }}^{\prime },\;\cdots ,\;\xi_{i_{q/2}^{\prime }}^{\prime }\right\}$ be the set of sequence value $\xi^{\prime }$ corresponding to sequence number $i_{\xi }^{\prime }$. The relationship among $\xi^{\prime },\;k^{\prime },\;t^{\prime }$ is $k^{\prime }=\xi^{\prime }\cdot \left(q^{2}+q+1\right)$ and $t^{\prime }=T\left(k^{\prime }\right)=T\left(\xi^{\prime }\cdot \left(q^{2}+q+1\right)\right)$. For $\forall \xi^{\prime }\in \Psi^{\prime }$, $m_{k^{\prime },0}\%2=0$ and $t^{\prime }=m_{k^{\prime },0}$ do not hold.

When ξ∈Ψ, one has $k=\xi (q^{2}+q+1)$ and $m_{k,0}\%2=0$, based on the Proposition 2, $t=m_{k,0}$ holds. According to Equation (22), the sequence number $i_{\xi }$ corresponding to sequence value ξ is given by $i_{\xi }=t\%q$. However, when $\xi^{\prime }\in \Psi^{\prime }$, one has $k^{\prime }=\xi^{\prime }\left(q^{2}+q+1\right)$ and $m_{k^{\prime },0}\%2\neq 0$, the Proposition 2 is not available, $t^{\prime }=m_{k^{\prime },0}$ does not hold. Therefore, the sequence number $i_{\xi^{\prime }}^{\prime }$ corresponding to sequence value $\xi^{\prime }\in \Psi^{\prime }$ cannot be determined by using Equation (22).

To further solve the above-mentioned problem, by selecting $k_{1}^{\prime },\;k_{2}^{\prime }\;(k_{1}^{\prime }\neq k_{2}^{\prime })$, one can obtain $m_{k_{1}^{\prime },0}$ corresponding to $k_{1}^{\prime }$, and $m_{k_{2}^{\prime },0}$ corresponding to $k_{2}^{\prime }$ by using chosen plaintext attack, which satisfies $m_{k_{1}^{\prime },0}\%2=1$ and $m_{k_{2}^{\prime },0}\%2=1$. Under this circumstance, although $T(k_{1}^{\prime })$ and $T(k_{2}^{\prime })$ are unknown, but according to the Proposition 2, ∀k corresponding to T(k)%2=0 can be found, so that the remained $\forall k^{\prime }$ satisfies $T(k_{1}^{\prime })\%2=1$ and $T(k_{2}^{\prime })\%2=1$, $|T\left(k_{1}^{\prime }\right)-T\left(k_{2}^{\prime }\right)|\%2=0$. According to the Corollary 1, it follows that

(23) $$m_{k_{1}^{\prime }k_{2}^{\prime },0}=|T\left(k_{1}^{\prime }\right)-T\left(k_{2}^{\prime }\right)\left|=\right|t_{1}^{\prime }-t_{2}^{\prime }|.$$

According to the chosen plaintext attack, $m_{k_{1}^{\prime }k_{2}^{\prime },0}$ in Equation (23) can be obtained from the given $\xi_{1}^{\prime },\;\xi_{2}^{\prime }\in \Psi^{\prime }$, where $\xi_{1}^{\prime }$ corresponding to $t_{1}^{\prime }$ satisfies $t_{1}^{\prime }=T\left(\xi_{1}^{\prime }\left(q^{2}+q+1\right)\right)$, and $\xi_{2}^{\prime }$ corresponding to $t_{2}^{\prime }$ satisfies $t_{2}^{\prime }=T\left(\xi_{2}^{\prime }\left(q^{2}+q+1\right)\right)$, respectively.

For the same $k_{1}^{\prime },\;k_{2}^{\prime }$, by selecting a suitable *k* such that $k=\xi (q^{2}+q+1)$, $m_{k,0}\%2=0$, one gets $\left[T\left(k_{1}^{\prime }\right)+T\left(k_{2}^{\prime }\right)-T\left(k\right)\right]\%2=0$. Then, according to the Corollary 2, it follows that

(24) $$m_{k_{1}^{\prime }k_{2}^{\prime }k,0}=T\left(k_{1}^{\prime }\right)+T\left(k_{2}^{\prime }\right)-T\left(k\right)=t_{1}^{\prime }+t_{2}^{\prime }-t,$$

where $T\left(k_{1}^{\prime }\right)<T\left(k\right)<T\left(k_{2}^{\prime }\right)$ or $T\left(k_{1}^{\prime }\right)>T\left(k\right)>T\left(k_{2}^{\prime }\right)$, $t_{1}^{\prime }<t<t_{2}^{\prime }$ or $t_{1}^{\prime }>t>t_{2}^{\prime }$.

According to the chosen plaintext attack, $m_{k_{1}^{\prime }k_{2}^{\prime }k,0}$ in Equation (24) can be obtained from the given $\xi_{1}^{\prime },\;\xi_{2}^{\prime }\in \Psi^{\prime }$ and ξ∈Ψ, where $\xi_{1}^{\prime }$ corresponding to $t_{1}^{\prime }$ satisfies $t_{1}^{\prime }=T\left(\xi_{1}^{\prime }\left(q^{2}+q+1\right)\right)$, $\xi_{2}^{\prime }$ corresponding to $t_{2}^{\prime }$ satisfies $t_{2}^{\prime }=T\left(\xi_{2}^{\prime }\left(q^{2}+q+1\right)\right)$, ξ corresponding to *t* satisfies $t=T\left(\xi \left(q^{2}+q+1\right)\right)=m_{k,0}$, in which $m_{k,0}$ is known by a chosen plaintext attack as well.

Note that one can also select $t_{i}^{\prime },t_{i+1}^{\prime },t\;(i=2,3,\cdots ,\left(q/2-1\right))$ in the same way, which is omitted here due to the limited length of the article.

According to Equations (23) and (24), four cases are given as follows:

(1) If $t_{1}^{\prime }<t<t_{2}^{\prime }$, then one has
  (25) $$\left\{\begin{array}{c} t_{2}^{\prime }=\left(m_{k_{1}^{\prime }k_{2}^{\prime },0}+m_{k_{1}^{\prime }k_{2}^{\prime }k,0}+t\right)/2=A_{1},\\ t_{1}^{\prime }=\left(-m_{k_{1}^{\prime }k_{2}^{\prime },0}+m_{k_{1}^{\prime }k_{2}^{\prime }k,0}+t\right)/2=B_{1}. \end{array}\right.$$
(2) If $t_{1}^{\prime }>t>t_{2}^{\prime }$, then one has
  (26) $$\left\{\begin{array}{c} t_{1}^{\prime }=\left(m_{k_{1}^{\prime }k_{2}^{\prime },0}+m_{k_{1}^{\prime }k_{2}^{\prime }k,0}+t\right)/2=A_{1},\\ t_{2}^{\prime }=\left(-m_{k_{1}^{\prime }k_{2}^{\prime },0}+m_{k_{1}^{\prime }k_{2}^{\prime }k,0}+t\right)/2=B_{1}. \end{array}\right.$$
(3) If $t_{2}^{\prime }<t<t_{3}^{\prime }$, then one has
  (27) $$\left\{\begin{array}{c} t_{3}^{\prime }=\left(m_{k_{2}^{\prime }k_{3}^{\prime },0}+m_{k_{2}^{\prime }k_{3}^{\prime }k,0}+t\right)/2=A_{2},\\ t_{2}^{\prime }=\left(-m_{k_{2}^{\prime }k_{3}^{\prime },0}+m_{k_{2}^{\prime }k_{3}^{\prime }k,0}+t\right)/2=B_{2}. \end{array}\right.$$
(4) If $t_{2}^{\prime }>t>t_{3}^{\prime }$, then one has
  (28) $$\left\{\begin{array}{c} t_{2}^{\prime }=\left(m_{k_{2}^{\prime }k_{3}^{\prime },0}+m_{k_{2}^{\prime }k_{3}^{\prime }k,0}+t\right)/2=A_{2},\\ t_{3}^{\prime }=\left(-m_{k_{2}^{\prime }k_{3}^{\prime },0}+m_{k_{2}^{\prime }k_{3}^{\prime }k,0}+t\right)/2=B_{2}. \end{array}\right.$$

Based on Equations (25)–(28), it follows that

(29) $$\left\{\begin{array}{c} \left\{t_{1}^{\prime },t_{2}^{\prime }\right\}=\left\{A_{1},B_{1}\right\},\\ \left\{t_{2}^{\prime },t_{3}^{\prime }\right\}=\left\{A_{2},B_{2}\right\}. \end{array}\right.$$

Then, according to Equation (29), it follows that

(30) $$\left\{\begin{array}{c} t_{2}^{\prime }=\left\{A_{1},B_{1}\right\}⋂\left\{A_{2},B_{2}\right\},\\ t_{1}^{\prime }=\left\{A_{1},B_{1}\right\}-\left\{t_{2}^{\prime }\right\},\\ t_{3}^{\prime }=\left\{A_{2},B_{2}\right\}-\left\{t_{2}^{\prime }\right\}. \end{array}\right.$$

Similarly, for $t_{i-1}^{\prime },t_{i}^{\prime },t$ and $t_{i}^{\prime },t_{i+1}^{\prime },t$, one has

(31) $$\left\{\begin{array}{c} t_{i}^{\prime }=\left\{A_{i-1},B_{i-1}\right\}⋂\left\{A_{i},B_{i}\right\},\\ t_{i-1}^{\prime }=\left\{A_{i-1},B_{i-1}\right\}-\left\{t_{i}^{\prime }\right\},\\ t_{i+1}^{\prime }=\left\{A_{i},B_{i}\right\}-\left\{t_{i}^{\prime }\right\}, \end{array}\right.$$

where i=2,3,⋯,(q/2−1).

For any given $\xi_{l}^{\prime }\in \Psi^{\prime }$ and ξ∈Ψ, according to Equation (31), first, one can get the corresponding $t_{l}^{\prime }$. Then, the sequence number $i_{\xi_{l}^{\prime }}^{\prime }$ corresponding to the sequence value $\xi_{l}^{\prime }$ can be further obtained by using $t_{l}^{\prime }$, such that

(32) $$\left\{\begin{array}{c} i_{\xi_{l}^{\prime }}^{\prime }=t_{l}^{\prime }\%q,\\ lx\left[i_{\xi_{l}^{\prime }}^{\prime }\right]=\xi_{l}^{\prime }, \end{array}\right.$$

where l∈{1,2,⋯,q/2}.

Finally, according to the Equations (22) and (32), one can determine all the sequence values ξ∈Ψ, $\xi_{l}^{\prime }\in \Psi^{\prime }$ and all the corresponding sequence numbers $i_{\xi }$, $i_{\xi^{\prime }}^{\prime }$ in Equation (2), so that the chaotic index sequence lx can be completely deciphered.

### 3.2. Analysis of Secret Keys α, β, γ

**Proposition** **3.** *Under the condition that the chaotic index sequence lx is obtained, for any $\left(l_{1},l_{2},l_{3}\right)\neq (l_{1}^{\prime },l_{2}^{\prime },l_{3}^{\prime })$, where $l_{i},l_{i}^{\prime }\in \left\{0,1,2,\cdots ,q-1\right\}\;(i=1,2,3)$, if $L_{1}\left(l_{1},l_{2},l_{3}\right)=L_{2}\left(l_{1},l_{2},l_{3}\right)\neq 0$ and $L_{2}\left(l_{1}^{\prime },l_{2}^{\prime },l_{3}^{\prime }\right)=L_{3}\left(l_{1}^{\prime },l_{2}^{\prime },l_{3}^{\prime }\right)\neq 0$, then the secret keys α,β,γ can be uniquely determined.*

**Proof.**  According to Equation (3), if $L_{1}\left(l_{1},l_{2},l_{3}\right)=L_{2}\left(l_{1},l_{2},l_{3}\right)\neq 0$ and $L_{2}\left(l_{1}^{\prime },l_{2}^{\prime },l_{3}^{\prime }\right)=L_{3}\left(l_{1}^{\prime },l_{2}^{\prime },l_{3}^{\prime }\right)\neq 0$ for any $\left(l_{1},l_{2},l_{3}\right)\neq (l_{1}^{\prime },l_{2}^{\prime },l_{3}^{\prime })$, then it follows that

(33) $$\left\{\begin{array}{c} L_{1}\left(l_{1},l_{2},l_{3}\right)=L_{2}\left(l_{1},l_{2},l_{3}\right)=c_{l_{1}}\times {\chi_{1}}^{2}+c_{l_{2}}\times \chi_{1}+c_{l_{3}}\neq 0,\\ L_{2}\left(l_{1}^{\prime },l_{2}^{\prime },l_{3}^{\prime }\right)=L_{3}\left(l_{1}^{\prime },l_{2}^{\prime },l_{3}^{\prime }\right)=c_{l_{1}^{\prime }}\times {\chi_{2}}^{2}+c_{l_{2}^{\prime }}\times \chi_{2}+c_{l_{3}^{\prime }}\neq 0, \end{array}\right.$$

where $c_{l_{1}},c_{l_{2}},c_{l_{3}}$ are sequence values of chaotic index sequence lx, $\chi_{1}\in \left\{\alpha ,\beta \right\}$, $\chi_{2}\in \left\{\beta ,\gamma \right\}$.

According to the first equation of Equation (33), one gets two solutions $\chi_{1}^{\left(1\right)},\chi_{1}^{\left(2\right)}$ for $\chi_{1}$. Similarly, according to the second equation of Equation (33), one gets two solutions $\chi_{2}^{\left(1\right)},\chi_{2}^{\left(2\right)}$ for $\chi_{2}$. Thus, there exists an intersection for the first equation and the second equation of Equation (33), given by $\beta =\left\{\chi_{1}^{\left(1\right)},\chi_{1}^{\left(2\right)}\right\}⋂\left\{\chi_{2}^{\left(1\right)},\chi_{2}^{\left(2\right)}\right\}$. Based on the deciphered secret key β, the remaining two secret keys $\alpha =\left\{\chi_{1}^{\left(1\right)},\chi_{1}^{\left(2\right)}\right\}-\left\{\beta \right\}$ and $\gamma =\left\{\chi_{2}^{\left(1\right)},\chi_{2}^{\left(2\right)}\right\}-\left\{\beta \right\}$ can further be deciphered as well.

If $L_{1}\left(l_{1},l_{2},l_{3}\right)=L_{2}\left(l_{1},l_{2},l_{3}\right)=0$ and $L_{2}\left(l_{1}^{\prime },l_{2}^{\prime },l_{3}^{\prime }\right)=L_{3}\left(l_{1}^{\prime },l_{2}^{\prime },l_{3}^{\prime }\right)=0$, then, an intersection for the first equation and the second equation of Equation (33) does not exist, so the secret keys α,β,γ cannot be obtained [39]. The proof is finished. □

### 3.3. Flowchart of Security Analysis

**Figure 5:**
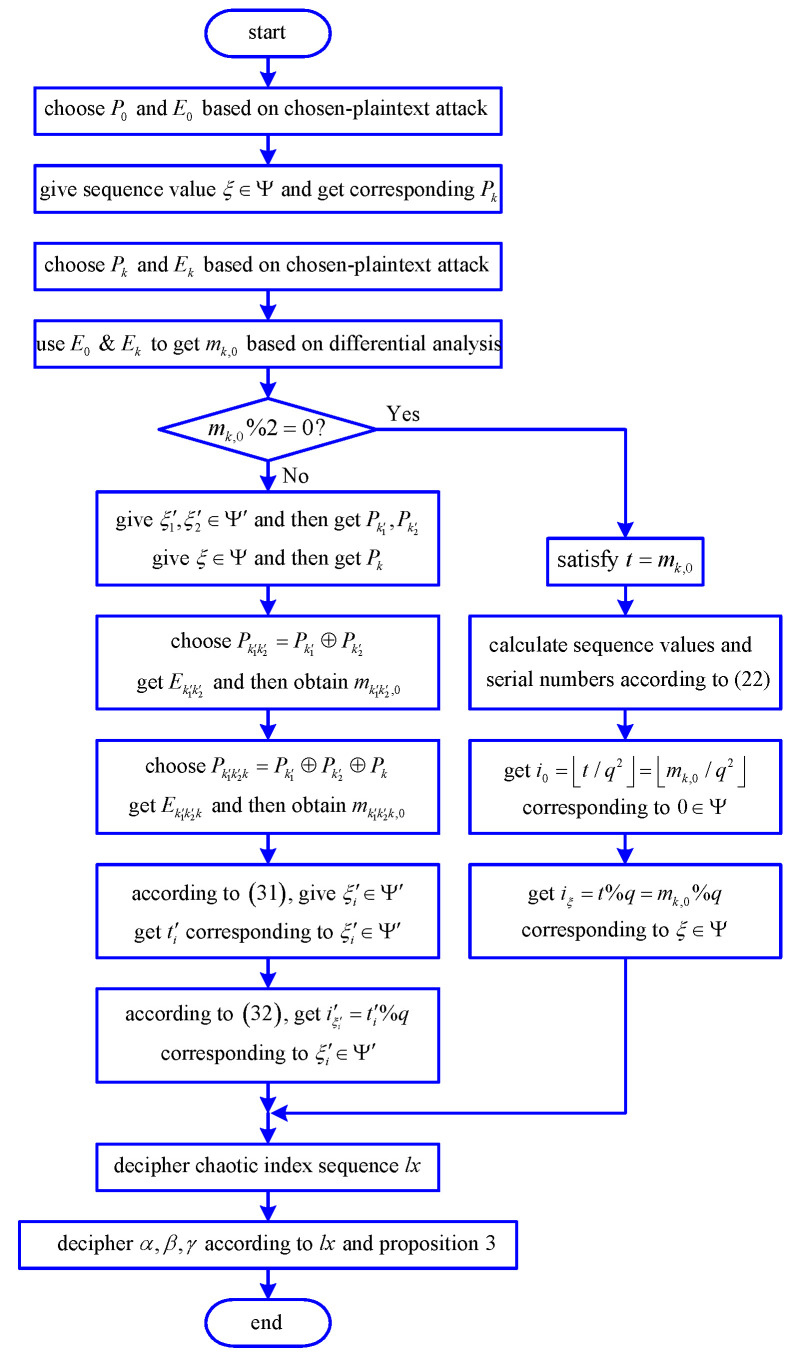
Flowchart of security analysis.

The flowchart of security analysis is shown in Figure 5.

## 4. Steps for Deciphering the Image Chaotic Encryption Algorithm

The steps for deciphering image chaotic encryption algorithm are as Algorithm 3.

**Algorithm 3** Steps for Deciphering Image Chaotic Encryption Algorithm.**Output:**    The equivalent secret keys lx,α,β,γ;
1:According to the chosen plaintext attack, choose the plain image as $P_{0}$, the corresponding cipher image is $E_{0}$, the 1D bit sequence corresponding to $E_{0}$ is ${\left\{e_{0}\left[i\right]\right\}}_{i=0}^{q^{3}-1}$.2:According to the chosen plaintext attack, choose the plain image as $P_{k}$, the corresponding cipher image is $E_{k}$, the 1D bit sequence corresponding to $E_{k}$ is ${\left\{e_{k}\left[i\right]\right\}}_{i=0}^{q^{3}-1}$. where $k=\xi \cdot (q^{2}+q+1)$, ξ∈Ψ.3:According to the differential attack, calculate $m_{k,0}$ by using ${\left\{e_{0}\left[i\right]\right\}}_{i=0}^{q^{3}-1}$ and ${\left\{e_{k}\left[i\right]\right\}}_{i=0}^{q^{3}-1}$ obtained in step 1 and step 2.4:If $m_{k,0}\%2=0$, then $t=m_{k,0}$ holds. According to Equation (22), the sequence number corresponding to sequence value 0 is $i_{0}=\left\lfloor t/q^{2}\right\rfloor =\left\lfloor m_{k,0}/q^{2}\right\rfloor$, the sequence number corresponding to sequence value ξ∈Ψ is $i_{\xi }=t\%q=m_{k,0}\%q$.5:If $m_{k}\%2=1$, then $t\neq m_{k,0}$ holds, Equation (22) is not available. According to the chosen plaintext attack, choose the plain image as $P_{k_{1}^{\prime }k_{2}^{\prime }}=P_{k_{1}^{\prime }}⊕P_{k_{2}^{\prime }}$, the corresponding cipher image is $E_{k_{1}^{\prime }k_{2}^{\prime }}$, the 1D bit sequence corresponding to $E_{k_{1}^{\prime }k_{2}^{\prime }}$ is ${\left\{e_{k_{1}^{\prime }k_{2}^{\prime }}\left[i\right]\right\}}_{i=0}^{q^{3}-1}$. In addition, choose the plain image as $P_{k_{1}^{\prime }k_{2}^{\prime }k}=P_{k_{1}^{\prime }}⊕P_{k_{2}^{\prime }}⊕P_{k}$, the corresponding cipher image is $E_{k_{1}^{\prime }k_{2}^{\prime }k}$, the 1D bit sequence corresponding to $E_{k_{1}^{\prime }k_{2}^{\prime }k}$ is ${\left\{e_{k_{1}^{\prime }k_{2}^{\prime }k}\left[i\right]\right\}}_{i=0}^{q^{3}-1}$.6:According to the differential attack, first calculate $m_{k_{1}^{\prime }k_{2}^{\prime },0}$ by using ${\left\{e_{0}\left[i\right]\right\}}_{i=0}^{q^{3}-1}$ and ${\left\{e_{k_{1}^{\prime }k_{2}^{\prime }}\left[i\right]\right\}}_{i=0}^{q^{3}-1}$ obtained in step 1 and step 5. Then, calculate $m_{k_{1}^{\prime }k_{2}^{\prime }k,0}$ by using ${\left\{e_{0}\left[i\right]\right\}}_{i=0}^{q^{3}-1}$ and ${\left\{e_{k_{1}^{\prime }k_{2}^{\prime }k}\left[i\right]\right\}}_{i=0}^{q^{3}-1}$ obtained in step 1 and step 5.7:According to Equation (32), calculate the sequence number $i_{\xi_{i}^{\prime }}^{\prime }=t_{i}^{\prime }\%q$ corresponding to sequence value $\xi_{i}^{\prime }\in \Psi^{\prime }$.8:Decipher the chaotic index sequence lx by using Equation (22) and Equation (32). Then, decipher the secret keys α,β,γ according to the Proposition 3.9:**return**lx,α,β,γ;


Theoretical analysis and experimental results indicate that only a maximum of $2.5\times \sqrt[{3}]{w\times h}$ plain images are needed to decipher the chaotic index sequence lx, and only a maximum of six plain images are needed to decipher secret keys α,β,γ. Therefore, only a maximum of $2.5\times \sqrt[{3}]{w\times h}+6$ is needed to crack the cipher image with w×h resolution.

## 5. Numerical Simulation Experiments

**Figure 6:**
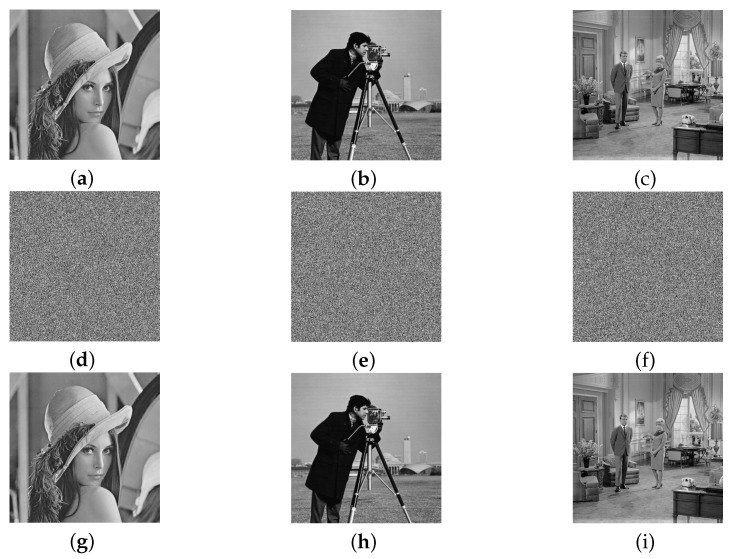
Plain images ((**a**–**c**) row), cipher images ((**d**–**f**) row), and deciphered images ((**g**–**i**) row) of Lena ((**a**–**g**) column), cameraman ((**b**–**h**) column), and living room ((**c**–**i**) column).

In the numerical simulation experiments, the secret keys are set as ${key}_{0}=0.34$, $\mu_{0}=3.9$, α=20, β=37, γ=46, the image is with 512×512 resolution. According to the steps for deciphering the image chaotic encryption algorithm given in Section 4, the deciphering algorithm of the origin cipher is implemented by the C program language. Simulations are operated under a laptop computer with Intel Core i7-8550U CPU (Santa Clara, CA, USA) 1.80 GHz, 8 GB RAM, the operating system is Microsoft Windows 10 (Redmond, WA, USA). Using the original algorithm to encrypt and use the algorithm proposed in this paper to crack an image with size of 512×512 takes about 0.115 s and 10.702 s, respectively. Since the encryption process of the algorithm is independent of plaintext and ciphertext, the equivalent key obtained by deciphering any ciphertext image can be used to decipher all ciphertext images of the same resolution. Taking the standard 2D plain gray image Lena, Cameraman, Livingroom as three examples, the plain images, the cipher images, and the deciphered images are shown in Figure 6, respectively.

**Figure 7:**
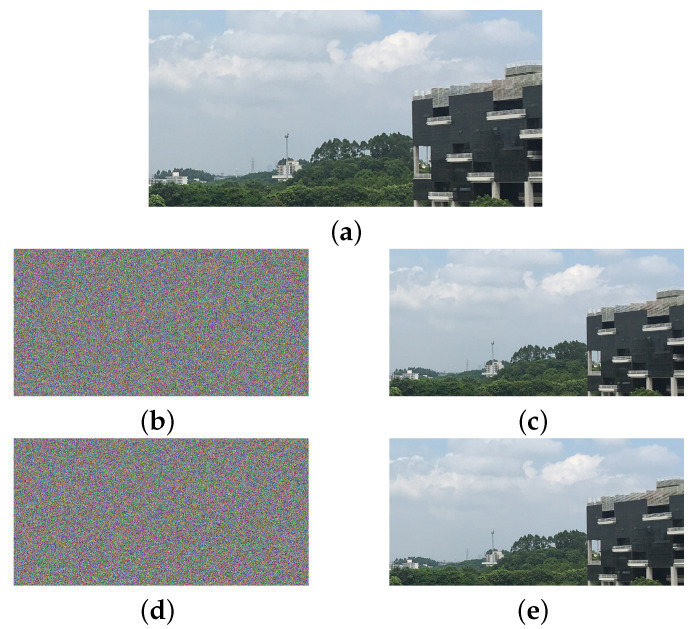
The result of the deciphering of the real-life image. (**a**) the original image; (**b**) encrypting the three color channels with the same key; (**c**) the deciphered image corresponding to (**b**); (**d**) encrypting the three color channels with three different sets of keys; (**e**) the deciphered image corresponding to (**d**).

Although the previous analysis is for grayscale images, the original encryption algorithm can be easily extended to encrypt color images by encrypting each of the three channels of the color image as a separate grayscale image. In this case, the attack method proposed in this paper is still valid. Take a real-life image with 1024×2048 resolution as an example. Encrypting this image using the original encryption algorithm, it takes about 0.53 s to encrypt the three color channels with the same key, and it takes about 107.36 s to decipher the corresponding ciphertext using the attack method proposed in this paper. Encrypting three color channels with three different sets of keys takes about 1.42 s, and it takes about 318.45 s to decipher the corresponding ciphertext. The results are shown in Figure 7.

## 6. Suggestions for Improvement

According to the analysis in Section 3, the original algorithm is insecure and cannot resist the choice of plaintext attack, and the complexity of the attack method is relatively low. To deal with its security defects, the corresponding suggestions for improvement to enhance the security are as follows:

(1) Enhance the sensitivity of the encryption algorithm to plaintext and ciphertext. According to the analysis in Section 3, the original algorithm has a universal equivalent key lx,α,β,γ. The original algorithm is not sensitive to both plaintext and ciphertext. The root cause of this defect is that the generation of Latin cubes is independent of plaintext image. This vulnerability can be solved by introducing some statistical properties of plaintext, such as the sum of all pixel values, into the generation phase of the Latin cubes.

(2) The mechanism used in the diffusion phase is too simple to achieve the avalanche effect of cryptography, which makes the encryption algorithm vulnerable to differential attacks. To fulfill this demand, increasing the number of encryption rounds or exploiting some complex diffusion mechanisms are worthy options.

## 7. Conclusions

This paper investigates the security of a Latin-bit cube-based image chaotic encryption algorithm. The algorithm adopts a first-scrambling-diffusion-second-scrambling three-stage encryption scheme. Although the designer claims that the algorithm has passed various statistical tests, the security analysis results in this paper demonstrate that the algorithm has some security vulnerabilities. In particular, the generation of Latin cubes is independent of plain image, and the change in the number of bits in the cipher image follows the change of any one bit in the plain image with obvious regularity. Thus, the equivalent secret keys lx,α,β,γ can be cracked by a chosen plaintext attack and differential attack. Only a maximum of $2.5\times \sqrt[{3}]{w\times h}+6$ plain images are needed to decipher the equivalent secret keys. Theoretical analysis and numerical simulation experiment results verify the effectiveness of the analytical method.
